# Sensor-Aided V2X Beam Tracking for Connected Automated Driving: Distributed Architecture and Processing Algorithms

**DOI:** 10.3390/s20123573

**Published:** 2020-06-24

**Authors:** Mattia Brambilla, Lorenzo Combi, Andrea Matera, Dario Tagliaferri, Monica Nicoli, Umberto Spagnolini

**Affiliations:** 1 Dipartimento di Elettronica, Informazione e Bioingegneria, Politecnico di Milano, 20133 Milan, Italy; lorenzo.combi@polimi.it (L.C.); andrea.matera@polimi.it (A.M.); dario.tagliaferri@polimi.it (D.T.); umberto.spagnolini@polimi.it (U.S.); 2 Dipartimento di Ingegneria Gestionale, Politecnico di Milano, 20156 Milan, Italy; monica.nicoli@polimi.it

**Keywords:** beam alignment, cooperative beam tracking, V2X, V2V, V2I, mmwave, free-space optics

## Abstract

This paper focuses on ultra-reliable low-latency Vehicle-to-Anything (V2X) communications able to meet the extreme requirements of high Levels of Automation (LoA) use cases. We introduce a system architecture and processing algorithms for the alignment of highly collimated V2X beams based either on millimeter-Wave (mmW) or Free-Space Optics (FSO). Beam-based V2X communications mainly suffer from blockage and pointing misalignment issues. This work focuses on the latter case, which is addressed by proposing a V2X architecture that enables a sensor-aided beam-tracking strategy to counteract the detrimental effect of vibrations and tilting dynamics. A parallel low-rate, low-latency, and reliable control link, in fact, is used to exchange data on vehicle kinematics (i.e., position and orientation) that assists the beam-pointing along the line-of-sight between V2X transceivers (i.e., the dominant multipath component for mmW, or the direct link for FSO). This link can be based on sub-6 GHz V2X communication, as in 5G frequency range 1 (FR1). Performance assessments are carried out to validate the robustness of the proposed methodology in coping with misalignment induced by vehicle dynamics. Numerical results show that highly directional mmW and/or FSO communications are promising candidates for massive data-rate vehicular communications even in high mobility scenarios.

## 1. Introduction

The technological development of connected, cooperative and automated systems is expected to represent a game-changer for mobility, with benefits for individual road users and effects on the societal impact in terms of sustainability and quality of life, changing the perspective of cities’ design [1,2,3]. Cooperative Intelligent Transportation Systems (C-ITS) are expected to improve the mobility experience in terms of efficiency, safety and comfort, breaking the conventional paradigm of human-controlled driving [4]. New technology is emerging in the context of the fifth Generation (5G) of cellular networks, which go beyond a simple upgrade of current mobile radio networks [5,6]. In the incoming years Vehicle-to-Anything (V2X) communications are required to guarantee fast sharing of massive mobility data through the vehicular cloud network, with unprecedented requirements on latency, data rate and reliability [7]. Examples include the exchange of raw (or partially processed) sensor data between vehicles to provide “extended sensors” functionalities for active safety applications such as “see-through” and “bird’s eye view”, enabling high Levels of Automation (LoAs). High-LoA services are based on the exchange of heavy data streams, coming from a huge number of different sensors that equip modern vehicles and in the order of tens/hundreds of Mbps each [8]. The demand of such services cannot be fulfilled by the currently available V2X technologies, namely the WiFi-based IEEE 802.11p [9] and the Cellular-V2X (C-V2X) [10]. These two standards are operating in the 5.9 GHz spectrum, as decided by the European Commission [11], providing interoperable and non-interfering C-ITS services targeted to improve safety in mobility [12], but they are not ready for the game-changing breakthrough of automated and connected automotive services. In this perspective, two partnerships, namely the Car 2 Car Communication Consortium (C2C-CC) [13] and the 5G Automotive Association (5GAA) [14], are being working towards the development of new standards specifically intended for the automotive vertical sector. The IEEE 802.11bd and 5G New Radio (NR) V2X standards are planned to fulfill the demand of high-LoA services [15,16], introducing operating capabilities at Millimeter-Wave (mmW) bands (as well as at sub-6 GHz).

The mmW radio is a viable candidate to match the challenging V2X requirements thanks to wide transmission bandwidth availability. The latter aspect is also experienced by considering Free-Space Optics (FSO) technology. As detailed later in the paper, although presenting several peculiarities, both technologies rely on near pencil propagating beams. This implies that both mmW and FSO need extremely precise Beam Alignment (BA), especially when the application involves high mobility. In this rapidly varying scenario, two main aspects limit high-speed V2X communications: Line-Of-Sight (LOS) blockage and beam-pointing misalignment. In this work, we focus on the latter problem, leaving the blockage analysis to future research with ad-hoc techniques. The following Section 1.1 and Section 1.2 discuss benefits and challenges of mmW and FSO V2X technologies, respectively, highlighting the original contribution of the proposed approach with respect to available literature. A more comprehensive and detailed description of contributions regarding the proposed sensor-aided BA and tracking procedure is provided in Section 1.3.

### 1.1. Millimeter-Wave V2X Communication

Today, mmW is considered the only viable Radio Frequency (RF) technology capable of satisfying the extreme latency and data-rate requirements of V2X communications thanks to the huge bandwidth available in this spectrum portion. Nevertheless, the use of mmW for enhanced V2X scenarios (eV2X) presents several challenges. First, high frequencies are subject to severe path loss, thus leading to a significant communication performance degradation due to atmospheric absorption and environmental obstructions. Mobility, Doppler effect, blockage and lack of context information are also critical issues that need to be properly addressed in the system design. In this regard, a promising solution is to deploy antenna arrays with many elements at both Transmitter (Tx) and Receiver (Rx) sides, i.e., massive Multiple-Input Multiple-Output (mMIMO) systems. The mMIMO technology allows shaping of multiple highly directive radiation beams in a confined spatial region, counteracting the severe path loss and minimizing the mutual interference. Furthermore, the reduced wavelength of mmW (10 mm at 30 GHz) allows packing hundreds of antennas in a small array, making mMIMO a viable technology for short-range (<1 km) smart-mobility applications.

Practical implementation of mmW mMIMO technology poses two main issues: (*i*) traditional MIMO systems require dedicated RF and baseband hardware at each antenna element to control signals’ amplitude/phase, and this is not viable for such a massive number of antennas, thus forcing mmW mMIMO systems to heavily rely on analog or RF processing; (*ii*) mmW mMIMO systems require precise BA and tracking procedures to keep the pencil beams aligned along the LOS direction in high mobility conditions. While lots of research efforts have been devoted to deal with the former issue [17,18,19,20,21], the latter is still open, and it is the main focus of this paper.

Conventional BA solutions that rely on an exhaustive search of the optimal Tx/Rx beam pair are too time demanding for vehicular scenarios due to the latency constraints. To speed up the BA procedure, different solutions have been proposed in the literature [22,23,24,25,26,27,28]. The authors of [29] propose to explore the channel and queue state information to optimize both transmission and reception beamwidths. Other promising approaches exploit side information to support the communication, such as location data provided by a radar signal operating in a different mmW band [30], motion prediction [31,32] or Global Positioning System (GPS) [33]. As detailed in the following, in this paper we follow an innovative approach, whereby BA and tracking are carried out based on the information retrieved from the on-board sensors that are mutually exchanged between vehicles through a parallel low-rate control link.

### 1.2. Free-Space Optics V2X Communication

FSO is a mature form of Optical Wireless Communication (OWC) in which the information exchange is through a modulated laser link, mostly operating in the visible or infrared part of the spectrum [34,35]. Although currently not standardized, FSO is envisioned for a wide range of applications: from high-capacity back-haul links for next generation cellular networks [36] to the interconnection between moving platforms such as Unmanned Aerial Vehicles (UAV), aircraft, satellites, trains and cars [37,38,39,40,41].

In the V2X context, FSO is interesting under many different aspects: (*i*) the huge optical bandwidth (currently unlicensed); (*ii*) the immunity from RF interference; (*iii*) the immunity from Doppler effect when inexpensive Intensity Modulation/Direct Detection (IM/DD) is employed; (*iv*) the availability of low-cost, small-size, high-speed integrated components and, more importantly, (*v*) the high spatial collimation of the beam that allows wide operational ranges (from a few meters to several kilometers depending on the visibility conditions) with low power expenditure and no interference between different FSO links in dense networks. Most of the benefits of FSO in terms of signal coverage come from the extremely directive beams employed for communication. However, FSO systems, besides being highly sensitive to atmospheric conditions such as turbidity (fog, haze, rain, etc.) and turbulence [42,43], are subject to frequent LOS loss due to random obstacles and, mostly, Tx-Rx misalignments. These arise from Tx/Rx vibrations, tilting and relative motion, which make the alignment a main challenge of FSO V2X communications, and this is covered here. The straightforward solution to cope with this issue is to widen the optical beam: this approach, although useful for very short-range systems [44], would lead to an unacceptable power density reduction at typical V2X distances, nullifying the advantages of a naively directive modulated laser beam. The most fruitful solution to exploit the high collimation of laser beams is, instead, to employ sophisticated Acquisition, Tracking, and Pointing (ATP) mechanisms to keep a seamless connection [45]. The FSO system must be able to first acquire the position of the receiver with a suitable spatial scanning phase, and successively to perform a continuous tracking of the receiver by a proper steering of the laser.

Numerous techniques have been proposed in the literature to address this issue [45]. In particular, Ref. [41,46] are particularly interesting for the purposes here as they provide an experimental proof-of-concept land-mobile FSO Vehicle-To-Infrastructure (V2I) system capable of transmitting information from a moving vehicle (max speed of 30 km/h) to a fixed receiver (road infrastructure) at a distance of approximately 1 km. The mobile transmitter knows the position of the fixed platform and combines it with its own location (using GPS) to coarsely steer the laser towards the receiver (acquisition), while the tracking is performed with the aid of two cameras and a beaconing system. Although interesting, [41,46] do not consider the more challenging Vehicle-to-Vehicle (V2V) scenario with higher mobility of all vehicles (i.e., ≫30 km/h) affected by tilting, in which the locations of Tx and Rx terminals cannot be known in advance. This is precisely the V2V context considered in this paper, which proposes to share information of the instantaneous dynamics of the vehicles by a data fusion of on-board sensor data. The exchange of pose data over a parallel low-rate control link provides each vehicle with a precise and timely information to be used for an accurate laser pointing.

### 1.3. Contribution: Sensor-Aided V2X Communication

Albeit presenting different peculiarities, both mmW and FSO leverage a V2X communication over narrow beams. Therefore, a common challenge is keeping a precise alignment between transmitter and receiver, and this is the focus of the paper. Conventional beam-tracking is based on various scanning strategies that are activated once the alignment is lost and the connection interrupted. Although providing precise results, these procedures involve a reduction of the communication efficiency due to the additional signaling overhead. Moreover, scanning is highly time-consuming, thus preventing the technologies at hand from meeting the V2X latency requirements [47]. In contrast, the solution proposed in this paper is a seamless BA strategy based on V2V sharing and distributed processing of sensor data. This article extends our previous work in [48], where a dual-layer system architecture for cooperative FSO beam-pointing is shown to provide enhanced V2V performance, by also combining map data and vehicle localization measurements. We extend the non-standalone V2X architecture with a sensor-aided control system for tracking highly directive V2X beams to the mmW case too, focusing on the extremely challenging V2V use case. Key feature of the proposed architecture is that the coexistence of two parallel communication technologies allows for the exchange of vehicle pose data on a low-rate control channel, such that the multi-gigabit link (either FSO- or mmW-based) is fully exploited to meet the stringent requirements of advanced C-ITS services and high-LoAs. The low-data-rate link (e.g., sub-6 GHz C-V2X or 5G NR Frequency Range (FR) 1) is only used for signaling vehicle dynamics information for BA control. This is in line with the 5G vision for V2X communications which foresees a multi-connectivity approach (non-standalone architecture [49]) to guarantee the reliability constraints of V2X Ultra-Reliable Low-Latency Communications (URLLC) through the combination of different communication modes (e.g., V2X and Vehicle-to-Network-to-Everything—V2N2X) or different Radio Access Technologies (RAT). In the proposed approach, the low-rate V2X link is a parallel control link used to improve the reliability of the high data-rate V2X link, and their combination provides the V2X URLLC.

Sensor data are typically collected in vehicles by on-board Inertial Measurement Units (IMU, including 3D accelerometers, 3D gyroscopes and magnetometers), along with camera systems, radar, Global Navigation Satellite System (GNSS) and other technologies. Presently, vehicles on the market use these heterogeneous sensors for driver assistance and partial automation applications. In this paper, we propose to process these sensor data not only to infer the ego vehicle dynamics, but primarily to extract information on the relative V2V dynamics by distributed processing of such data over the vehicular cloud. Seamless BA and tracking is designed based on the exchange of dynamic pose information (position and orientation) between the networked vehicles. Sensing and communications are tightly coupled in the proposed architecture, as on-board sensors are exploited to augment the V2X communication performance, just as V2X is used to extend the ego-sensing capability.

The paper contributes and structure can be summarized as follows. We first introduce a C-ITS architecture to enable highly directive (i.e., mmW- or FSO-based) V2X communications in which the pose of the V2X terminal of the ego vehicle is estimated by fusing data from different on-board sensors accounting for their temporal variability. Then we detail a sharing mechanism of the ego-pose information among all the connected V2X terminals (i.e., among the vehicles) through a parallel low-rate, low-latency signaling channel, so that each terminal has a complete knowledge of the overall system geometry. Lastly we analyze the feasibility of the proposed high data-rate mmW- and FSO-based V2X solutions by performance assessments on a realistic 3D modeling of the vehicles’ dynamics in a challenging V2V scenario, analyzing the Cumulative Distribution Function (CDF) of the Signal-to-Noise Ratio (SNR) both for mmW and FSO. The performance assessment is entirely focused on the impact of vehicle dynamics on the beam-pointing. Despite describing a V2V-specific system architecture (which is the most challenging one due to the high mobility of both Tx and Rx terminals), the proposed methodology also applies to any V2X communication system with straightforward adaptations. For example, it can be easily extended to V2I communications, in which one Tx/Rx terminal is fixed and, thus, it is not required to continuously estimate its pose.

Some preliminary results of the proposed approach herein proposed can be found in [48,50,51]. The works [50,51], however, rely on two different system models defined ad-hoc for 2-vehicle communications (the model in [50] is limited to 2D geometry) and, more importantly, both lack of a global vision on the system architecture enabling the proposed V2X communication. The work in [48], on the other hand, focuses only on FSO technology. Differently from the former works, in this paper we present a C-ITS architecture which complies with both mmW and FSO V2X technologies under a unified framework. The model in [50,51] is extended to include position and angular estimation errors, and it now takes into account an arbitrary number of vehicles. Furthermore, the unified model allows for a performance comparison between mmW and FSO, which spots pros and cons of both technologies and emphasizes which of the two is more suitable for any specific V2X scenario.

### 1.4. Organization

The paper is organized as follows: the overall V2X architecture is described in Section 2, while the formalization of the 3D geometry of the problem is in Section 3. Section 4 presents the channel models for the mmW and FSO communications, whereas Section 5 shows some numerical results. Section 6 draws the conclusions and summarizes the challenges that are still to be faced.

### 1.5. Notation

Bold upper- and lower-case letters describe matrices and column vectors. Matrix transposition is indicated as ${\left(\cdot \right)}^{T}$ while the Hermitian adjoint ${\left(\cdot \right)}^{H}$. $I_{m}$ denotes the identity matrix of size *m*. R is the symbol for the set of real numbers, while C for the complex ones. Operator ||·|| represents the Euclidean norm and, lastly, operator ∘ stands for the Hadamard product.

## 2. Proposed V2X System Architecture

**Figure 1:**
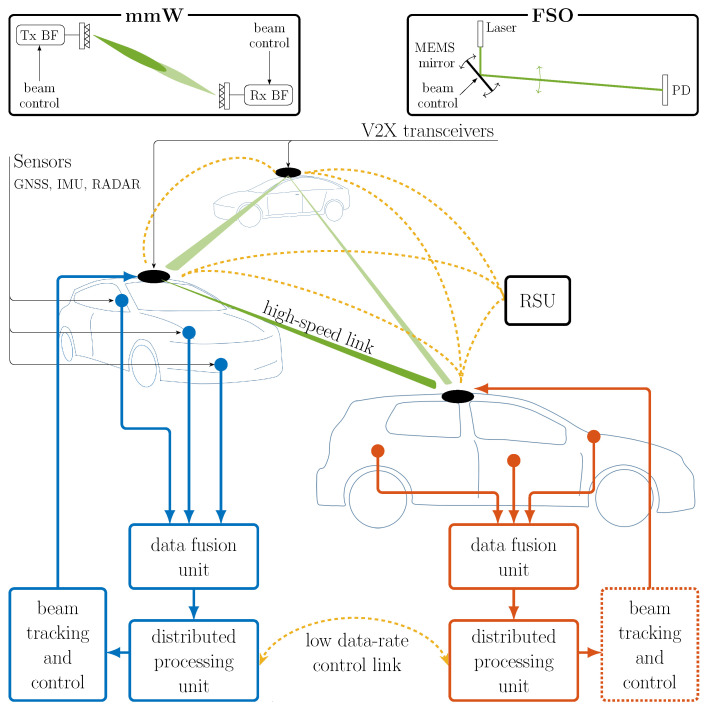
Overview of the proposed sensor-aided cooperative V2X architecture.

The envisioned V2X architecture is represented in Figure 1. This comprises several inter-connected vehicles, which are assumed to be fully equipped with many different sensors such as IMU, cameras, GNSS, radar, LiDAR and others. The Road-Side Units (RSUs), whenever existing, enhance the V2V network performance, e.g., by providing internet connection, forwarding control messages in case of Non-Line-Of-Sight (NLOS) conditions, providing updated 3D road maps. The role of the RSU can be covered by the radio access network such as 5G macro/micro cells. The main feature of the proposed architecture is the two-layer V2X communication, which operate in parallel to provide (*i*) a high data-rate link (either mmW or FSO) for high-LoA scenarios (green beams in Figure 1) and (*ii*) a low-data-rate link (either V2V or V2I) for exchanging locally processed information about vehicles’ position and orientation (yellow arcs in Figure 1).

As stressed in Section 1.3, mmW and FSO require advanced beam-tracking mechanisms to comply with the reliability and latency requirements of V2X communications, and this is where the need for a low-rate, low-latency, dedicated control link comes from. As shown in Figure 1, at first each vehicle fuses the data from the many various on-board sensors in order to estimate/predict the temporal evolution of its own pose (data fusion unit). Then, through the aforementioned low-rate control link, the pose data are shared among all vehicles in the network. The distributed processing unit of each vehicle has then an accurate knowledge of the V2X network geometry, consisting of the position and orientation in space and time of all the V2X agents (vehicles and possibly RSUs). In this way, all agents have the information needed to compute the time evolution of the beam-pointing directions, providing superior FSO- and mmW-based V2X communication performance.

The time-varying vehicle pose is typically affected by vibrations and tilting, and it can be modeled as the superimposition of a low-pass random process, modeling the “smooth” motion of the vehicle (e.g., a sharp turn, or a high-speed curve that induces a roll rotation), and a fast-varying random process induced by road roughness, bumps, etc. Their combination induces a time variability of the vehicle pose that requires a fast tracking of beam-pointing directions to steer the V2X beams. In this scenario, also the sensors’ sampling frequency and their accuracy, as well as the latency on the low-data-rate link play a major role to guarantee the robustness and reliability of the whole systems. Comments and modeling details on these issues are given in the following sections.

## 3. Vehicle Dynamics Modeling and Beam-Pointing

The goal of this section is to detail the 3D geometrical model for V2V communications generalized to an arbitrary number of vehicles and capable of describing the alignment for both mmW and FSO V2V communication scenarios. A correct alignment of the beams relies on the knowledge of the relative pose (3D position and 3D orientation) of the Tx and Rx vehicles. Each vehicle is thus described by 6 Degrees of Freedom (DoFs), collecting its position and orientation with respect to a local navigation frame that is Earth-fixed and common to all vehicles in the networks.

**Figure 2:**
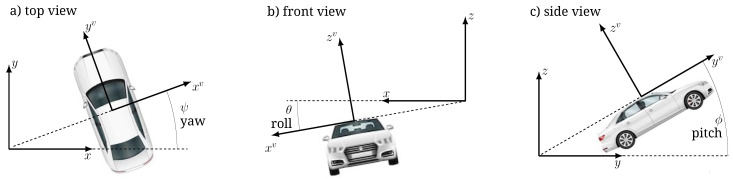
Navigation and vehicle frames showing the separate effect of the three Cardan angles. (**a**) top view with $\phi_{v},\theta_{v}=0,\psi_{v}\neq 0$; (**b**) front view with $\phi_{v},\psi_{v}=0,\theta_{v}\neq 0$; (**c**) side view with $\theta_{v},\psi_{v}=0,\phi_{v}\neq 0$.

**Figure 3:**
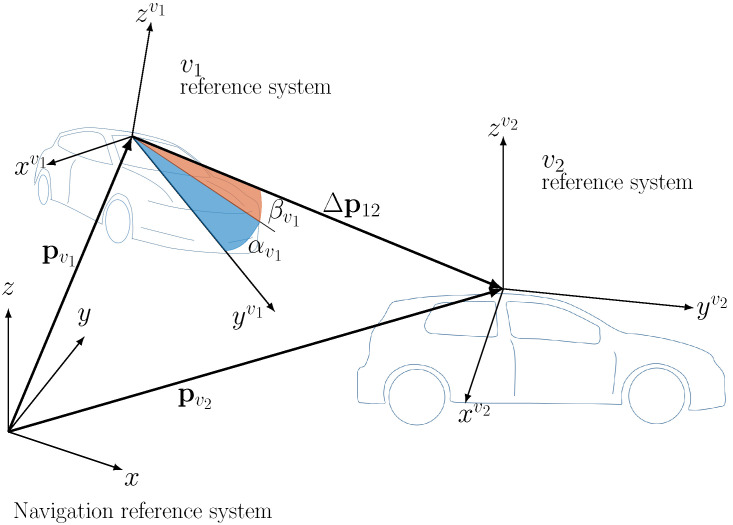
Geometry for a 2-vehicles network, with navigation and vehicle frames and LOS angles for Tx vehicle $v_{1}$.

In this direction, the problem at hand is two-fold: at each time instant we first need to determine the pose of each vehicle with respect to the common local navigation reference system, and then to derive the pointing directions in each individual vehicle reference system. These directions are the input to the beam-pointing that is composed by Tx and Rx beamformers for mmW, and by a Micro Electro-Mechanical System (MEMS) mirror at the Tx side for FSO. As shown in Figure 2 and Figure 3, the reference systems are defined as follows: the local navigation reference system is fixed with respect to the Earth, centered in a suitable point to describe the local vehicular network, and with the *x* axis pointing towards East, *y* axis towards North and *z* axis towards the sky. Each vehicle reference system, identified by the superscript *v*, is fixed with respect to the corresponding vehicle, centered at the mmW/FSO transceiver (that we place on top of the vehicle), and with $y^{v}$ axis pointing towards the front bumper, $x^{v}$ to the right, $z^{v}$ towards the sky.

### 3.1. Vehicle Dynamics Modeling

The dynamics of each vehicle (which is modeled as a rigid body in this paper), is uniquely described by its position vector with respect to the navigation reference system $p_{v}\left(t\right)={\left[p_{v,x}\left(t\right)\;p_{v,y}\left(t\right)\;p_{v,z}\left(t\right)\right]}^{T}\in R^{3\times 1}$ and its orientation $\gamma_{v}\left(t\right)$. The latter can be represented in many different ways such as quaternion notation or composing three elemental rotations. Here we use the Cardan angles convention, leading to the orientation vector

(1) $$\gamma_{v}\left(t\right)={\left[\begin{array}{ccc} \phi_{v}\left(t\right) & \theta_{v}\left(t\right) & \psi_{v}\left(t\right) \end{array}\right]}^{T}\;$$

that collects the pitch, roll and yaw, respectively. The reference system of vehicle *v* is therefore obtained from a roto-translation of the navigation reference: a translation by $p_{v}\left(t\right)$ composed with a rotation described by the matrix $R^{v}\left(\gamma_{v}\left(t\right)\right)$. This matrix is the result of three successive rotations around *x* (by $\phi_{v}\left(t\right)$), *y* (by $\theta_{v}\left(t\right)$) and *z* (by $\psi_{v}\left(t\right)$), and its final expression (for a given time *t*, thus omitted) is [52]:

(2) $$R^{v}\left(\gamma_{v}\right)=\left[\begin{array}{ccc} \cos\theta_{v}\cos\psi_{v} & -\cos\theta_{v}\sin\psi_{v} & -\sin\theta_{v}\\ \cos\phi_{v}\sin\psi_{v}-\sin\phi_{v}\cos\psi_{v}\sin\theta_{v} & \cos\phi_{v}\cos\psi_{v}-\sin\phi_{v}\sin\theta_{v}\cos\psi_{v} & -\sin\phi_{v}\cos\theta_{v}\\ \sin\phi_{v}\sin\psi_{v}+\cos\phi_{v}\cos\psi_{v}\sin\theta_{v} & \sin\phi_{v}\cos\psi_{v}+\cos\phi_{v}\sin\theta_{v}\cos\psi_{v} & \cos\phi_{v}\cos\theta_{v} \end{array}\right]$$

The dynamics of vehicle *v* as a rigid body is then described by 6 DoFs, summarized by the position $p_{v}\left(t\right)$ and the Cardan angles $\gamma_{v}\left(t\right)$ with respect to an absolute reference system. The separate effect of the three Cardan angles on the vehicle orientation with respect to the navigation frame is in Figure 2. These position and orientation vector are now used to derive the optima LOS direction of V2V communication link.

### 3.2. Derivation of Line-of-Sight Direction

Let us consider the communication between two vehicles within the network as in Figure 3, in which $v_{1}$ is the transmitter and $v_{2}$ is the receiver: the pose of the two vehicles is described by the two positions $p_{v_{1}}\left(t\right)$ and $p_{v_{2}}\left(t\right)$ in the navigation reference system, and the two rotation matrices $R^{v_{1}}\left(t\right)=R^{v}\left(\gamma_{v_{1}}\left(t\right)\right)$ and $R^{v_{2}}\left(t\right)=R^{v}\left(\gamma_{v_{2}}\left(t\right)\right)$.

At the transmitter side ($v_{1}$), the V2V distance vector is

(3) $$\Delta p_{21}^{v_{1}}\left(t\right)=R^{v_{1}}\left(t\right)\left(p_{v_{2}}\left(t\right)-p_{v_{1}}\left(t\right)\right)={\left[\begin{array}{ccc} \Delta p_{21,x}^{v_{1}}\left(t\right) & \Delta p_{21,y}^{v_{1}}\left(t\right) & \Delta p_{21,z}^{v_{1}}\left(t\right) \end{array}\right]}^{T}$$

and the LOS direction for vehicle $v_{1}$ is identified by the azimuth angle $\alpha_{v_{1}}\left(t\right)$ and the elevation angle $\beta_{v_{1}}\left(t\right)$, respectively defined as:

(4) $$\begin{array}{c} \alpha_{v_{1}}\left(t\right)=\mathrm{atan}\frac{\Delta p_{21,x}^{v_{1}}\left(t\right)}{\Delta p_{21,y}^{v_{1}}\left(t\right)}\;\;\;,\;\;\;\beta_{v_{1}}\left(t\right)=\mathrm{asin}\frac{\Delta p_{21,z}^{v_{1}}\left(t\right)}{∥\Delta p_{21}^{v_{1}}∥}\; \end{array}$$

where the operator $∥\cdot ∥$ denotes the Euclidean norm. Similarly, at the receiver side, the vector connecting the two vehicles is $\Delta p_{12}^{v_{2}}\left(t\right)=R^{v_{2}}\left(t\right)\left(p_{v_{1}}\left(t\right)-p_{v_{2}}\left(t\right)\right)$, and the computation of the pointing angles $\alpha_{v_{2}}\left(t\right)$ and $\beta_{v_{2}}\left(t\right)$ directly follows from Equation (4) with straightforward modifications.

### 3.3. Estimation of Line-of-Sight Direction

Ideally, the best communication performance is obtained when the Tx and Rx are always perfectly aligned along the LOS direction. This means that for mmW, the radiating beams of both Tx and Rx vehicles point towards the LOS angles in Equation (4) for every time *t*, while for FSO that the Tx laser is always perfectly illuminating the Rx photodiode (or array of photodiodes). In practice, the knowledge of the optimal time-varying pointing angles (corresponding to the LOS directions) is a crucial issue, and the update rate of the pointing directions is a key design parameter for the system. This problem is addressed here by a mutual exchange of pose information between vehicles, which is obtained by a local processing of on-board sensors’ measurements.

Pose estimation consists of evaluating the dynamics over time of each vehicle frame (orientation and position) with respect to the navigation frame, which represents a highly challenging task. As a matter of fact, GNSS typically provides a position estimate that is not accurate enough for eV2X applications. Among the augmentation possibilities, refinement using 5G cellular data has been proposed [53], as well as leveraging an implicit localization with respect to fixed features (e.g., streetlamps, traffic lights, tollbooths, etc.) [54,55]. Concerning the orientation estimation, gyroscope, accelerometer and magnetometer data from the IMU can be integrated, provided that smart techniques are used to handle integration drift [56]. Measurement and dynamic models can be included in an extended Kalman filtering approach, possibly also combining camera and radar data [52].

Position and orientation estimates for each vehicle are thus affected by various noise sources yielding to noisy pose estimates as:

(5) $$\begin{array}{cc} {\hat{p}}_{v}\left(t\right) & =p_{v}\left(t\right)+w_{p,v}\left(t\right)\;, \end{array}$$

(6) $$\begin{array}{cc} {\hat{\gamma }}_{v}\left(t\right) & =\gamma_{v}\left(t\right)+w_{\gamma ,v}\left(t\right)\;, \end{array}$$

where $w_{p,v}∼N\left(0,C_{p}\right)$ and $w_{\gamma ,v}∼N\left(0,C_{\gamma }\right)$ can be modeled as stationary Gaussian processes [52]. It is to be noticed that the errors on Cardan angles are in general mutually correlated. The same holds for the errors on position and orientation, as these are jointly obtained by a pose estimator, and the estimation of LOS angles, i.e., the outcome of the BA, is from the pose of at least two different vehicles. A thorough characterization of these stochastic terms is beyond the scope of this work.

## 4. Communication System Model

In the following, we first describe how the proposed V2X architecture fits into standardized communication protocols (Section 4.1 and Section 4.2), and then detail the peculiarities of the mmW and FSO communication channels in Section 4.3 and Section 4.4, respectively.

### 4.1. Conventional Time-Slotted Frame Structure

The following description is intended to provide a generalized overview of a Time Division Duplex (TDD) frame, without referring to any specific existing protocol. The frame structures of the IEEE 802.11ad standards and the draft of IEEE 802.11ay amendment [57,58] are used as references, since they are both tailored for mmW communications operating at 60 GHz, even if originally intended for stationary/quasi-stationary applications. Since no communication protocol has been standardized yet for FSO communications [59], we assume here that the reference frame structure adopted for mmW is suitable for FSO as well. It is, however, worth noticing that it is straightforward to extend the discussion to a time-slotted frequency division duplex protocol.

**Figure 4:**
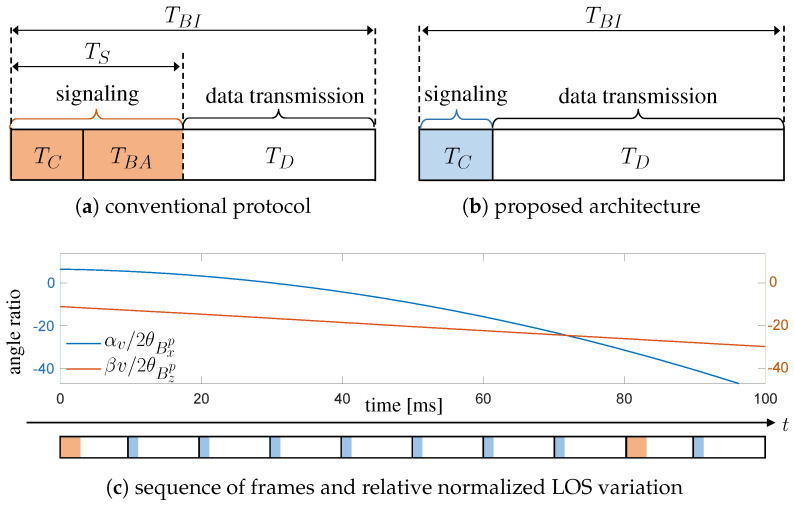
TDD frame structure. (**a**) a conventional protocol with BA included in the signaling; (**b**) the proposed architecture; (**c**) Sequence of frames and an example of variation of pointing angles $\alpha_{v}$ and $\beta_{v}$ in a typical vehicular scenario, normalized with respect to a beamwidth of $2\theta_{B_{x}^{p}}=2\theta_{B_{z}^{p}}=0.1$ deg (an achievable value for FSO systems). The frame duration is chosen $T_{BI}=10$ ms to match 5G specifications.

As a general rule, the TDD communication frame comprises two main access periods: the first is allocated to the exchange of signaling information related to protocol and network management, the latter to data transmission. In this way, the whole beacon interval of duration $T_{BI}$ consists of a signaling interval ($T_{S}$), which is further subdivided into general control signaling ($T_{C}$) and BA-specific signaling ($T_{BA}$), and a data transmission interval ($T_{D}$), as illustrated in Figure 4a.

When dealing with highly directional communications, most of signaling is intended for the implementation of beam alignment/tracking procedures which are time-consuming, especially in vehicular scenarios [33]. In conventional BA techniques, Tx and Rx discover the best direction of transmission using a closed-loop beam training strategy, based on testing some predefined beam patterns that could have different resolutions. The overall transmission efficiency η is given by the ratio of the time interval dedicated to data transfer over the whole beacon interval as:

(7) $$\eta_{\mathrm{conv}}=1-\frac{T_{C}+T_{BA}}{T_{BI}}\;.$$

From Equation (7) it is straightforward to conclude that a reduction of $T_{BA}$ would significantly increase the communication efficiency, which is even worse for multiple vehicles. To this aim, the next section is fully dedicated to describing how the proposed V2X architecture addresses this issue and suggests an alternative frame structure.

### 4.2. Proposed Frame Structure and Beam Alignment Procedure

In the proposed architecture, the beam alignment is aided by the vehicle pose estimated from sensor data. Indeed, the angles $\alpha_{v}\left(t\right)$ and $\beta_{v}\left(t\right)$ in Equation (4) describing the LOS direction evolve over time according to the dynamics of the vehicles in the network $(p_{v}\left(t\right)$ and $\gamma_{v}\left(t\right))$. Exchanging the pose estimates through the low-rate control link allows updating of the beam-pointing direction virtually without any beam alignment signaling, and the resulting transmission efficiency would be

(8) $$\eta =\frac{1-\frac{T_{C}}{T_{BI}}}{1+\frac{R_{L}}{R_{H}}}\approx 1-\frac{T_{C}}{T_{BI}}\;,$$

where $R_{H}\gg R_{L}$ are the data rates on the high-speed (mmW or FSO) V2X link and on the low-rate parallel control link, respectively. This reduction is highlighted in the frame representation in Figure 4b.

On the other hand, the proposed sensor-based beam-tracking opens the following two issues: the update rate of the beam-pointing with respect to the frame duration and the reliability of the geometry-based beam-pointing alone. As far as the first issue is concerned, the latency of the parallel control link plays a main role in determining the accuracy of pose information. A high latency, in fact, would be detrimental as the exchanged information easily becomes outdated. Regarding the second issue, instead, the technical characteristics of sensors are pivotal to provide detailed measurements of vehicle position ${\hat{p}}_{v}\left(t\right)$ and orientation ${\hat{\gamma }}_{v}\left(t\right)$. In fact, the pose information is updated with a frequency given by the sampling rate of sensor data. If 1 kHz update is deemed adequate for the V2X application at hand, and assuming 16-bit quantization of the 6 pose DoFs, each vehicle generates a 96-kbps stream on the control link. If a more frequent update is necessary for the specific V2X BA application, the data rate on the control link increases still satisfying the condition $R_{L}\ll R_{H}$.

Basing the beam-pointing solely on the estimated dynamics of vehicles makes the whole high-speed V2X link prone to errors in position and orientation estimation. To improve systems robustness in this respect, we envision a system that includes the presence, every once in a while, of a frame with the conventional beam alignment procedure. In these settings, the conventional beam alignment can benefit from the existence of a prior beam direction estimated from the system geometry. This sequence of transmission frames is sketched in Figure 4c, together with the variation of the pointing angles in a typical vehicular scenario (details in Section 5). In particular, Figure 4c shows the variation in pointing directions normalized to a typical beamwidth of a FSO system ($2\theta_{B_{x}^{p}}$ and $2\theta_{B_{z}^{p}}$, better defined in Equation (22) in Section 4.4.2), which highlights the need for a frequent update of the beam-pointing.

As the overhead introduced by conventional protocol is inefficient for a multi-gigabit V2X communication, in the proposed V2X architecture, the period between two consecutive conventional BA frames (in which a search of the optimal pointing angles is carried out) is extended by introducing a series of BA-free frames with meaningful reduction of overhead. Clearly, the overall transmission efficiency depends on both types of frame, but an evaluation of the optimal combination is left to future research activities.

### 4.3. Millimeter-Wave V2V

The V2V mmW scenario considered in this subsection includes two vehicles that communicate through a mmW mMIMO LOS communication link. The goal is to define a mathematical model for the mmW channel, as well as the array geometry, to evaluate the impact on the V2V transmission capacity of the mismatch between the LOS and the estimated beam-pointing directions (i.e., ${\left[\alpha_{v},\;\beta_{v}\right]}^{T}$ and ${\left[{\hat{\alpha }}_{v},\;{\hat{\beta }}_{v}\right]}^{T}$) at vehicles $v_{1}$ and $v_{2}$.

#### 4.3.1. Cylindrical Array Geometry

**Figure 5:**
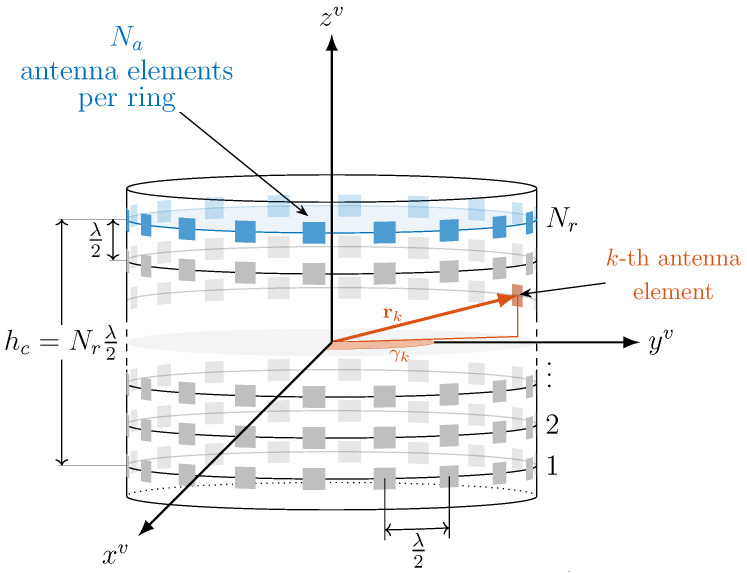
Cylindrical array for mmW V2X.

Differently from the simple 2D mmW case presented in [50], where the analysis is limited to a Uniform Linear Array (ULA), this paper considers a more realistic 3D mmW communication scenario in which each vehicle is assumed to be equipped with a conformal array (such as a 3D cylindrical array) to guarantee an isotropic radiation. This is depicted in Figure 5, and it is composed of $N_{r}$ rings with $N_{a}$ antennas each, i.e., $N=N_{r}N_{a}$ antenna elements overall. This choice of array is led by the application, where the Tx and Rx can be randomly displaced all over the horizontal plane. Thus, a circular symmetric array design allows an array gain that is independent from the pointing direction.

To compute the steering vector for the considered cylindrical array, it is first necessary to define the position vector $r_{k}^{v}$ of the *k*-th antenna element in the vehicle reference system, which is given by the array radius *r*, the array height $h_{c}$ and the angular position $\gamma_{k}$ of the *k*-th antenna element. Assuming the peripheral distance between two adjacent antenna elements belonging to the same ring equal to $\frac{\lambda }{2}$ (with λ denoting the carrier wavelength), the radius of the cylindrical array yields $r=\frac{1}{2\pi }\frac{\lambda }{2}N_{a}$, where $\frac{\lambda }{2}N_{a}$ is the ring perimeter. Similarly, considering half-wavelength-spaced rings, the array height yields $h_{c}=\frac{\lambda }{2}N_{r}$, and the angular position of the *k*-th antenna element of each ring is $\gamma_{k}=\frac{k}{N_{a}}2\pi -\pi$. It follows that locating the origin of the reference system in the center of the array, the position vector $r_{k}^{v}$ for the *k*-th antenna element can be computed as:

(9) $$r_{k}^{v}={\left[\begin{array}{c} r\cos\gamma_{k}\;\;\;\;\;\;r\sin\gamma_{k}\;\;\;\;\;\;-\frac{h_{c}}{2}+\left\lceil \frac{k}{N_{a}}\right\rceil \frac{\lambda }{2} \end{array}\right]}^{T},$$

as illustrated in Figure 5. Given the pair of pointing angles ($\alpha_{v}$, $\beta_{v}$) of vehicle *v*, the steering vector $\bar{a}\left(\alpha_{v},\beta_{v}\right)\in C^{1\times N}$ for the cylindrical array with omnidirectional antenna elements is thus defined as:

(10) $$\bar{a}\left(\alpha_{v},\beta_{v}\right)={\left[e^{-\frac{j2\pi }{\lambda }r_{1}^{v^{T}}u^{v}}\;\;\;\;\cdots \;\;\;\;e^{-\frac{j2\pi }{\lambda }r_{N}^{v^{T}}u^{v}}\right]}^{T},$$

where

(11) $$u^{v}={\left[\begin{array}{c} \cos\beta_{v}\sin\alpha_{v}\;\;\;\;\;\;\cos\beta_{v}\cos\alpha_{v}\;\;\;\;\;\;\sin\beta_{v} \end{array}\right]}^{T}$$

identifies the instantaneous wave propagation direction in the vehicle reference system.

The steering vector in Equation (10) denotes a conventional beamforming technique which is used by Tx/Rx vehicle to irradiate/capture the electromagnetic energy in/from a confined spatial region for omnidirectional pattern of the antenna elements [60]. Clearly, this model does not perfectly match a commercial hardware and its adoption could lead to inaccurate results. For this reason, along with the steering vector, we introduce a directivity vector $D(\alpha_{v},\beta_{v})$, where the *k*-th entry is computed (under far-field assumption) as [61]:

(12) $$\begin{array}{c} D_{k}\left(\alpha_{v},\beta_{v}\right)=b\cos^{c}\left(\min(|\alpha_{v}-\gamma_{k}|,\pi \;/\;2)\right)\cos^{c}\left(\beta_{v}\right)\;, \end{array}$$

providing a cosinusoidal pattern (on both azimuth and elevation) and baffling the back of each element. (Please note that the analysis of different tapering or processing techniques of the array vector is out of the scope of this work, despite significant variations on the overall antenna array gain can be experienced.). The resulting cylindrical array response is thus:

(13) $$\begin{array}{c} a\left(\alpha_{v},\beta_{v}\right)=D\left(\alpha_{v},\beta_{v}\right)\circ \bar{a}\left(\alpha_{v},\beta_{v}\right)\;. \end{array}$$

In this way, along the LOS direction, the overall gain $G_{v}$ of the cylindrical array is a function of the beam-pointing direction $\left({\hat{\alpha }}_{v},{\hat{\beta }}_{v}\right)$ and it is computed as:

(14) $$G_{v}=G\left({\hat{\alpha }}_{v},{\hat{\beta }}_{v}|\alpha_{v},\beta_{v}\right)=\frac{1}{N}|a^{H}\left({\hat{\alpha }}_{v},{\hat{\beta }}_{v}\right)a\left(\alpha_{v},\beta_{v}\right){|}^{2}.$$

The gain in Equation (14) is maximum when the pointing and LOS directions coincide. However, unavoidable errors in the knowledge of the instantaneous pose (mainly due to mobility) lead to a beam-pointing mismatch that reduces the antenna gains $G_{v}$ and can completely hinder the communication. This problem needs to be handled also in the proposed architecture where vehicles share information on a low-rate link, as the latency makes the information slightly obsolete. We will consider this effect later on in Section 5, showing how the relatively slow evolution of vehicle dynamics with respect to communication delay impact on the V2V performance. In addition, among all parameters impacting a V2V link budget, $G_{v}$ is the most affected by errors on the knowledge of the reciprocal pose, thus demonstrating that the occurrence of moderate antenna gain losses is a key aspect for the assessment of the V2V mmW capacity, which is derived in the next section.

#### 4.3.2. Millimeter-Wave V2V Performance

The used metric for performance evaluation is the SNR, defined as the ratio between the signal and the noise powers at the receiver:

(15) $${\mathrm{SNR}}^{\mathrm{mmW}}=\frac{P_{\mathrm{rx}}^{\mathrm{mmW}}}{P_{\mathrm{noise}}}\;.$$

The received power $P_{\mathrm{rx}}$ is influenced by the transmit power $P_{\mathrm{tx}}$, the antenna gains at both Tx and Rx sides, $G_{1}$ and $G_{2}$, respectively, and the signal path loss $PL^{\mathrm{dB}}$. The latter is computed as

(16) $$PL^{\mathrm{dB}}=20\log_{10}\left(\frac{4\pi }{\lambda }\right)+10\;\kappa \;\log_{10}∥\Delta p_{21}^{v_{1}}∥+\chi_{sh}\;,$$

where κ is the path loss exponent and $\chi_{sh}∼N\left(0,\sigma_{\mathrm{dB}}^{2}\right)$ is the log-normal distributed shadowing [62]. Thus, the received power (in dBm) can be expressed as

(17) $$P_{\mathrm{rx}}^{\mathrm{dBm}}=P_{\mathrm{tx}}^{\mathrm{dBm}}+G_{v_{1}}^{\mathrm{dB}}+G_{v_{2}}^{\mathrm{dB}}-PL^{\mathrm{dB}}.$$

On the other hand, the noise power $P_{\mathrm{noise}}$ at the receiver is evaluated as

(18) $$P_{\mathrm{noise}}^{\mathrm{dBm}}=N_{fl}+10\log_{10}B+NF\;,$$

where $N_{fl}$ is the noise floor, *B* is the signal bandwidth and NF is the noise figure. The system capacity is evaluated as

(19) $$C^{\mathrm{mmW}}=B\log\left(1+{\mathrm{SNR}}^{\mathrm{mmW}}\right)\;,$$

and the maximum effective data rate of the V2V link is

(20) $$R^{\mathrm{mmW}}=\eta C^{\mathrm{mmW}}\;.$$

### 4.4. Free-Space Optics V2V

As with the mmW case, this section describes the laser and photodetectors arrangements for the proposed FSO-based V2X communication.

#### 4.4.1. Laser and Photodetector Circular Array Geometry

**Figure 6:**
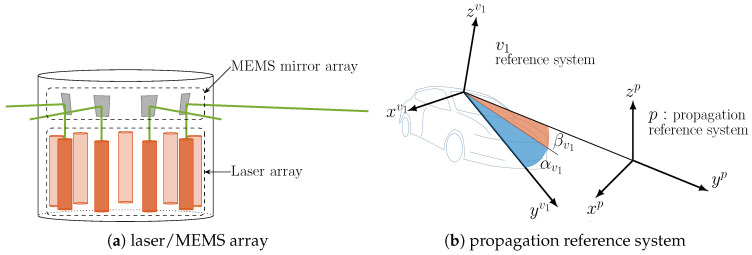
FSO V2V system. (**a**) laser/MEMS array for FSO V2X. (**b**) propagation reference system for the description of the laser beam, translated along the pointing direction for aesthetic purposes.

While the mmW beam steering procedure is well known, changing the pointing direction of a laser beam involves a completely different technology. Among the available ones, we consider to employ a four-quadrant MEMS Fast Steering Mirror (FSM), which provides the best trade-off among angular range (typically, ±10 degrees in both horizontal and vertical directions), resolution (sub-μrad), integrability, reduced size and power consumption [45]. Since, in general, horizontal variations of the pointing are much larger than the vertical ones due to the relative drifting of the two vehicles during their motion, we assume the presence of multiple laser beams placed in a circular-like array (as in Figure 6a) to guarantee a complete coverage, similarly to the cylindrical antenna array outlined in the previous subsection. The sizing of the angular spacing must enable the continuous sampling of the azimuth direction, and must account for the MEMS’ steering capability. Here we use 18 laser-mirror couples that are independently controlled to transmit up to 18 V2X data streams. These can be reduced to 8 laser-mirror pairs if a wide-angle lens is employed (±22.5 degrees scanning capability [63]). Dually, the receivers are configured to be isotropic by a cylindrical PD array, where each PD is equipped with a proper telescope (or, in general, a set of focusing lenses to increase the received signal). In this way there is always one or more PDs of the receiving unit oriented towards the transmitting laser to guarantee the Tx-Rx alignment.

#### 4.4.2. Free-Space Optics V2V Performance

As first assessment of the link budget, we consider an FSO transmission between one laser and one or more PDs belonging to the receiving unit, operating in clear sky conditions (neither turbidity nor turbulence), without obstacles. As common to standard single-mode laser diodes, the emitted signal can be well approximated by a Gaussian beam (first Transverse Electromagnetic mode, TEM${}_{00}$), whose propagation along a given direction is described by diffraction theory [64]. Differently from the mmW case, the analytic description of the laser beam propagation is greatly simplified by the use of a Tx-based reference system in which one of the axis is pointed along the direction of propagation, as shown in Figure 6b. By virtue of the geometrical model described in Section 3, this can be obtained by rotating the $v_{1}$ reference system according to the estimated pointing angles ${\left[{\hat{\alpha }}_{v_{1}},\;{\hat{\beta }}_{v_{1}}\right]}^{T}$, i.e., by applying the following rotation matrix

(21) $$R^{pv_{1}}=R_{x}\left({\hat{\beta }}_{v_{1}}\right)R_{z}\left({\hat{\alpha }}_{v_{1}}\right)\;,$$

where the superscript *p* indicates an additional propagation reference system used to describe the laser beam. The new axes are referred to as $\left(x^{p},y^{p},z^{p}\right)$, where $y^{p}$ identifies the location of maximum intensity of the beam, which has a Gaussian decay in the transversal directions ($x^{p}$, $z^{p}$). In this framework, the divergence half angles along the transversal $x^{p}$ and $z^{p}$ coordinates are equal to:

(22) $$\begin{array}{c} \theta_{B_{x}^{p}}=\frac{\lambda }{\pi W_{0_{x^{p}}}}\;\;\;,\;\;\;\theta_{B_{z}^{p}}=\frac{\lambda }{\pi W_{0_{z^{p}}}}\;, \end{array}$$

where λ is the wavelength of the transmitted lightwave, $W_{0_{x^{p}}}$ and $W_{0_{z^{p}}}$ are the beam waist sizes (minimum widths) of the beam along $x^{p}$ and $z^{p}$, respectively, defining an ellipse on the plane normal to the laser direction enclosing 86.5% of the transmitted power $P_{\mathrm{tx}}$. Without loss of generality, we can assume that the beam waist of the laser beam is located at the transmitting side, resulting in the Gaussian approximation for the beam intensity at distance *d*:

(23) $$I\left(d,x^{p},y^{p}\right)=\frac{2P_{\mathrm{tx}}}{\pi W_{x^{p}}\left(d\right)W_{z^{p}}\left(d\right)}\;\exp\left(-\frac{2{\left(x^{p}\right)}^{2}}{W_{x^{p}}\left(d\right)}\right)\exp\left(-\frac{2{\left(z^{p}\right)}^{2}}{W_{z^{p}}\left(d\right)}\right)\;,$$

where the transversal power decay is controlled by the spot size parameters

(24) $$\begin{array}{c} W_{x^{p}}\left(d\right)=W_{0_{x^{p}}}\;\sqrt{1+{\left(\frac{\lambda \;d}{\pi W_{0_{x^{p}}}^{2}}\right)}^{2}}\approx \theta_{B_{x^{p}}}d\;\;\;,\;\;\;W_{z^{p}}\left(d\right)=W_{0_{z^{p}}}\;\sqrt{1+{\left(\frac{\lambda \;d}{\pi W_{0_{z^{p}}}^{2}}\right)}^{2}}\approx \theta_{B_{z^{p}}}d\;, \end{array}$$

that are linearly dependent on *d* if $d>\pi W_{0_{x^{p}/z^{p}}}^{2}/\lambda$ (far-field condition). The optical signal is then captured by all the PDs that are in visibility with the laser. The received power at each PD is function of both the effective area $A_{\mathrm{rx}}$, and of the position of the receiver with respect to the laser spot. This is defined by the distance (Δx,Δz along the coordinates $x^{p},z^{p}$) between the PD and the center of the laser beam, as a result of a pointing misalignment ($\alpha_{v_{1}}-{\hat{\alpha }}_{v_{1}}$, $\beta_{v_{1}}-{\hat{\beta }}_{v_{1}}$). Furthermore, the non-orthogonal incidence of the laser beam onto the PD is represented by two angles ($\beta_{v_{2}}$ and $\zeta_{v_{2}}$). The overall effect is to project $A_{\mathrm{rx}}$ in the Rx-Tx direction.

The link budget between the Tx and the single PD is obtained by integrating the received power density as expressed in Equation (23) over the collecting aperture:

(25) $$P_{\mathrm{rx}}^{\mathrm{FSO}}\approx I\left(d,\Delta x,\Delta z\right)A_{rx}\cdot \cos\beta_{v_{2}}\cdot \cos\zeta_{v_{2}}\;,$$

where the approximation holds for the receiver collection area much smaller than the laser spot, i.e., $A_{\mathrm{rx}}\ll W_{x^{p}}\left(d\right)\;W_{z^{p}}\left(d\right)$. The displacements can be computed as (paraxial approximation):

(26) $$\begin{array}{c} \Delta x≃d\tan\left(\alpha_{v_{1}}-{\hat{\alpha }}_{v_{1}}\right)\;\;\;,\;\;\;\Delta z≃d\tan\left(\beta_{v_{1}}-{\hat{\beta }}_{v_{1}}\;\right)\;. \end{array}$$

Finally, the electrical SNR for an IM/DD transmission and Positive-Intrinsic-Negative (PIN)-based PDs is obtained by summing up all the power received by each single element. The final expression is [44,64]:

(27) $${\mathrm{SNR}}^{\mathrm{FSO}}=\frac{\sum_{k=1}^{N_{PD}}{\left(\rho P_{\mathrm{rx},k}^{\mathrm{FSO}}\right)}^{2}}{2\;e\;B\left(\underset{I_{b}}{\underset{︸}{E_{b}\;\Delta \lambda }}\;N_{PD}A_{\mathrm{rx}}\;\rho +\sum_{k=1}^{N_{PD}}\rho P_{\mathrm{rx},k}^{\mathrm{FSO}}\;\right)+{\mathrm{NEP}}^{2}\;\rho^{2}\;B}\;,$$

where:

- $N_{PD}$ denotes the number of PDs on which the signal impinges;
- The numerator is the sum of the squared electrical currents produced by the signal incident on each PD, with a responsivity of ρ;
- The first term at the denominator is the shot noise associated with the background light-induced current (i.e., the solar radiation), and to the useful signal. Symbol *e* denotes the electron’s charge. The solar irradiance $I_{b}$$\left[W/m^{2}\right]$ is assumed to be isotropic and it is obtained by multiplying the spectrum $E_{b}$$\left[W/m^{2}/\mathrm{nm}\right]$ and the receiver’s optical bandwidth Δλ (limited by the responsivity or by a proper optical filter);
- The second term at the denominator is the current noise power comprising both the dark current of the photodetector and the overall electronic noise generated by the receiving circuitry (mostly from the first amplifying stage). It is summarized by the input-referred Noise Equivalent Power NEP $\left[W/\sqrt{\mathrm{Hz}}\right]$.

As the employed IM/DD transmission techniques is based on real-valued only constellation symbols, the data rate is now computed as:

(28) $$R^{\mathrm{FSO}}=\eta \frac{B}{2}\;\log\left(1+{\mathrm{SNR}}^{\mathrm{FSO}}\right)\;.$$

## 5. Numerical Results

In this work, we evaluate and compare the performance of the proposed V2X solution based on either mmW or FSO technologies in terms of SNR, service probability $P_{S}$ and Fade Duration Distribution (FDD). It is important to mention that the results are obtained under two different degrees of beam directivity, i.e., *High Directivity (HD)* and *Low Directivity (LD)*, respectively. However, investigating two different technologies that have specific and sensibly different physical properties, it happens that a low directivity for FSO is considered to be high for mmW, since it is relatively easy to manipulate the optical beam. Nonetheless, we adopted, to the best of our abilities, comparable simulation parameters for mmW and FSO communication links, to guarantee a fair performance comparison. The common simulation parameters are in Table 1, together with main settings of mmW and FSO technologies.

### 5.1. Simulated Vehicular Scenario

Vehicles $v_{1}$ and $v_{2}$ are assumed to travel close each other as in a platoon formation. To evaluate the robustness of each technology and the impact of vehicles’ spatial dynamics, two completely different vehicular scenarios (namely S1 and S2) have been considered, differentiating the modeling of p(t). In particular, S1 represents a platooning scenario with vehicles that move along a straight road with constant inter-distance and mutual dynamics only on the vertical axis z (due to vibrations and tilting), while S2 describes a more complex trajectory with curves and height changes, so that the motion varies on all axes.

For each scenario, we define a position vector (expressed in the navigation frame)

(29) $$\begin{array}{c} p\left(t\right)=\left\{\begin{array}{c} {\left[\;vt\;\;\;\;\;\;0\;\;\;\;\;\;0\;\right]}^{T}\;\mathrm{for}\;S1,\;\\ {\left[r_{0}\left(1\;+\;A_{h}\cos\left(\omega_{h}t\right)\right)\cos\left(\omega_{0}t\right)\;\;\;\;\;r_{0}\left(1\;+\;A_{h}\cos\left(\omega_{h}t\right)\right)\sin\left(\omega_{0}t\right)\;\;\;\;\;A_{v}\cos\left(\omega_{v}t\right)\right]}^{T}\;\mathrm{for}\;S2, \end{array}\right. \end{array}$$

where the trajectories’ parameters are in Table 1. The 3D vehicle position for $v_{1}$ is modeled as:

(30) $$\begin{array}{c} p_{v_{1}}\left(t\right)=p\left(t\right)+{\left[0\;\;\;\;\;\;0\;\;\;\;\;\;h_{v_{1}}+\delta z_{v_{1}}\left(t\right)\right]}^{T}\;, \end{array}$$

where the term $h_{v_{1}}$ defines the vehicle height with respect to the road pavement and $\delta z_{v_{1}}\left(t\right)$ accounts for the vehicle stroke (calibrated on measured data [50,51]). Similarly, the position of $v_{v_{2}}$ is:

(31) $$\begin{array}{c} p_{v_{2}}\left(t\right)=p\left(t-\Delta t\right)+{\left[0\;\;\;\;\;\;0\;\;\;\;\;\;h_{v_{2}}+\delta z_{v_{2}}\left(t\right)\right]}^{T}, \end{array}$$

where Δt stands for the vehicle time gap between $v_{1}$ and $v_{2}$.

The quality and timeliness of the pointing information exchanged between the two vehicles across the parallel low-rate control link depend on two critical parameters: the sensors sampling rate $f_{\mathrm{data}}$ and the delay τ (mainly due to the end-to-end latency over the low-rate control link). This last parameter, in fact, introduces a delay in the update of pointing angles ($\alpha_{v}$, $\beta_{v}$) leading to an incorrect BA. Here we assume that the sampling rate is fixed to $f_{\mathrm{data}}=1$ kHz that implies the use of high-performance IMUs, and τ∈[1,15] ms. The choice of this value of delay is led by considering the targeted performance of 5G (and beyond) systems which are planned to provide ultra-low-latency (<1 ms) communications, while a more realistic short-term hardware foresees a delay in the order of 10 ms. The signal bandwidth *B* and the Tx power are set to B=2.16 GHz [57] and $P_{\mathrm{tx}}=1$ mW (0 dBm) for both mmW and FSO.

Moreover, to better evaluate the mmW and FSO robustness with respect to pose estimation errors, the covariances of measurement noise in Equations (5) and (6) are set to

(32) $$\begin{array}{c} R_{p}=\sigma_{p}^{2}I_{3}\;\;\;,\;\;\;R_{\gamma }=\sigma_{\gamma }^{2}I_{3}\;, \end{array}$$

with $\sigma_{p}=10$ cm and $\sigma_{\gamma }=0.1$ deg, unless otherwise specified. The position accuracy $\sigma_{p}$ is chosen as to meet the 5G service requirements for eV2X scenarios [47]. The angular accuracy can be achieved by very accurate state of the art automotive IMU or by averaging multiple on-board sensors or advanced data fusion techniques.

### 5.2. Millimeter-Wave Settings

**Figure 7:**
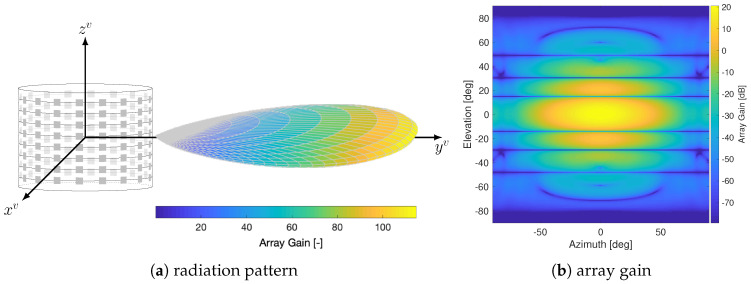
Array pattern for *mmW LD* pointing towards broadside direction (α=0 deg, β=0 deg): (**a**) tridimensional representation of the radiation pattern and (**b**) the array gain in dB towards different azimuth and elevation angles.

In this work we assume a mmW V2X link operating at 60 GHz carrier frequency. To fairly compare mmW and FSO and analyze the impact of vehicle dynamics and vibrations (we recall that a characterization of blockage is out of the scope of this work), we shall consider a scenario where no obstacles are present within the LOS path, for which a FSO system would be in outage. For this reason, we assume to have a free-space propagation (i.e., κ=2 and $\chi_{sh}=0$), and the interaction of the propagating wave with the vehicle roof is neglected. The choice of LOS V2V link is led by the need to isolate and evaluate the impact of vehicle motion and vibrations, ignoring additional sources of perturbation that are typical of a communication link (such as shadowing, fading, blockage or interference). The cylindrical array configuration (defined by the number of rings $N_{r}$ and antenna elements of each ring $N_{a}$) described in Section 4.3.1 changes across simulations in order to provide performance results under different degrees of beam directivity. In particular, we consider two mmW configurations described in Table 2. The first configuration, *mmW LD* (with N=256 antenna elements) represents a mmW solution that is not extremely directive and which implementation is feasible considering today’s hardware limitations. The second one (*mmW HD*), instead, considers an overly directive beam obtained with *N* = 16,200. This second sample scenario allows us, first, to evaluate the potential that mmW could achieve with prospective hardware technology and, secondly, to have a beam dimension closer to the narrow-laser FSO solution, and so to have meaningful comparisons. An example of array directivity for *mmW LD* is illustrated in Figure 7, for pointing directions coinciding with LOS at broadside ($\alpha_{v}=0\;\deg,\beta_{v}=0\;\deg$).

### 5.3. Free-Space Optics Settings

The performance of the FSO V2V link is evaluated here in clear sky conditions, in order to focus only on the impact of beam misalignments and to avoid the loss by adverse weather conditions (this is beyond the scope of the paper and is left as future research activity). We consider a Tx laser at λ=1550 nm, for which there is a large availability of high-speed integrated Distributed Feed-Back (DFB) sources with emitting power ranging from fractions to a few mW (eye-safe lasers [66]). As done for mmW, we simulate two configurations of the FSO system, reported in Table 2. Our aim is to investigate the performance and the requirements of the system in case of extremely narrow beam widths that are hardly obtainable with RF antenna arrays. Since it is relatively easy to obtain large directivity for FSO systems (by employing mm-size lenses), we explore divergences down to a full angle of 0.1 deg. The resulting laser spot diameters at their waist is bounded to be less than 2 mm, a value compatible with integrated MEMS-based steering mirrors [63]. Once more, as for mmW, we neglect the interaction of the laser beam with the car roof. To collect the largest possible fraction of power while limiting as much as possible the extra-size and weight of the system, we assume the cylindrical array of receivers to be of 10 cm diameter and 5 cm height. Each single receiver comprises a GHz-bandwidth InGaAs PIN PD covered by a focusing telescope. The outer diameter (sensible area) of each single receiving unit ($A_{\mathrm{rx}}$) is set to 1 cm${}^{2}$. We evaluate the background light for a vertical surface in the worst possible case, i.e., when both the direct sunlight and the skylight are maximum. Therefore, we consider the solar spectrum obtained for the geographical area of Milan, on 20 July 2018, at 12 a.m., assuming a very clear day (see [51] for details). Finally, the receiver is also equipped with an optical filter with a bandwidth of 50 nm [67], centered around 1550 nm, in charge of reducing the background light.

### 5.4. Performance Evaluation in Two Distinct Vehicular Scenarios

This section presents the performance evaluation of the proposed sensor-aided V2X beam-tracking obtained in the two different driving scenarios. The transmission scheme for the proposed method considers a frame structure as in Figure 4b for the whole simulation, meaning that the system fully relies on sensor’s information and never performs an exhaustive search of the optimal beam. Results are presented in terms of CDF of the SNR, service probability and FDD. We choose the CDF rather than the average SNR as it allows understanding of the range of achieved values, providing an assessment of the V2V outage. In safety critical V2X applications, the peak (or the average) value of SNR and, in turn, of the data rate represents only one quality indicator. The timeliness in providing information is another key performance indicator that must be considered in the assessment as any delay in communication or data-rate drop could have a severe impact on road user safety. For this reason, it is also important to analyze the FDD as it characterizes the duration of outage periods, where the SNR persists below a given threshold Γ and, thus, the communication is prevented.

**Figure 8:**
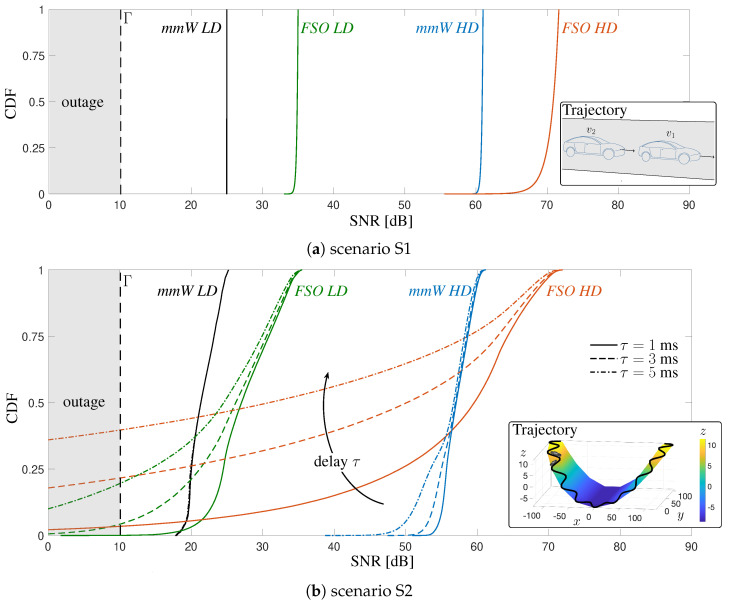
CDF of the SNR for the proposed sensor-aided beam-tracking method in two different scenarios. Results are plotted for both mmW and FSO *LD/HD* configurations for three different values of delay τ (vehicle time gap Δt=1 s). In the inset, part of the trajectory is shown.

The CDFs of mmW and FSO V2V systems, in both configurations and scenarios, are illustrated in Figure 8, for a vehicle time gap Δt=1 s.

By this result, besides providing insights on the maximum achievable data rate and the average one, we want to analyze the impact of delay on the V2V link as well as the type of trajectory and geometry. Referring to Figure 8a, we demonstrate that in a scenario where the mutual vehicle dynamics occurs only along the *z* axis, meaning that vehicles oscillate around the height $h_{v}$ at rest condition, it is possible to have a reliable V2V communication both at mmW and FSO: the SNR is almost constant for any *HD* and *LD* configuration, only for *FSO HD* a slight degradation is present, due to the extreme directivity of the laser. Moreover, in these settings, no performance degradation has been experienced because of a delay in updating the pointing parameters caused by latency. On the other hand, results in Figure 8b indicate that the type of trajectory and, in turn, the relative geometry, can play a major role in determining the quality of the V2V link. In fact, although the peak values of SNR are the same as in S1, in S2 the CDFs present broader tails, especially for FSO, confirming that the vehicle motion can easily lead to misalignment conditions that induce a SNR degradation on the V2V link. This reduction in SNR is directly related to the beam dimension and the timeliness of the shared information. Indeed, a significant worsening of the SNR is experienced by increasing the delay and narrowing the beam dimension, as expected.

**Figure 9:**
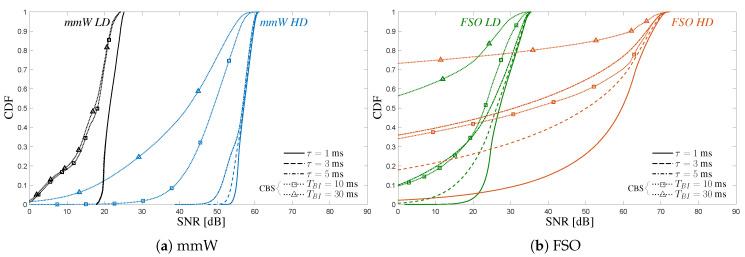
CDF of the SNR in scenario S2: comparison of the proposed sensor-aided beam-tracking method with a conventional beam sweeping (CBS) approach (markers). For CBS, frame durations of 10 and 30 ms are considered.

In the challenging scenario S2, it also interesting to compare the performance of the proposed sensor-aided beam-tracking method with a Conventional Beam Sweeping (CBS) procedure. CBS periodically performs an exhaustive search over pre-determined equispaced spatial sectors scanning all the horizontal and vertical dimensions. The periodicity coincides with the frame duration $T_{BI}$ (see Figure 4a), while the spatial spacing is given by the system resolution on both azimuth ($\Delta \alpha_{-3\mathrm{dB}}$ or $2\theta_{B_{x}^{p}}$) and elevation ($\Delta \beta_{-3\mathrm{dB}}$ or $2\theta_{B_{z}^{p}}$). To this extent, two different $T_{BI}$ are considered: 10 ms (as in 5G specifications) and 30 ms [33]. Results are shown in Figure 9 for both mmW (Figure 9a) and FSO (Figure 9b) technologies.

The comparison highlights how the proposed sensor-aided tracking allows for remarkable improvements in terms of SNR, especially for mmW. It is important to mention that the considered CBS simulation assumes that the exhaustive search is instantaneously completed. This aspect, in practice, is a major impairment for the feasibility of CBS. Taking the mmW case as example, equispaced steering vectors consider a uniform sampling of the azimuth and elevation, leading to several spatial sectors proportional to the square of antenna elements (i.e., $O{\left(N_{a}N_{r}\right)}^{2}$). The latest 5G New Radio standard foresees a search over up to 64 sectors per frame. On one side, this limitation poses significant limitations to CBS with mMIMO systems (or, in general, narrow beam systems), on the other calls for new BA strategies. This loss of efficiency is a main motivation behind the proposed integration of sensors’ information in the BA and tracking process which, besides providing improvements to the absolute value of SNR, also improves the channel capacity by significantly increasing the efficiency of the mmW/FSO link (see Section 4.1).

**Figure 10:**
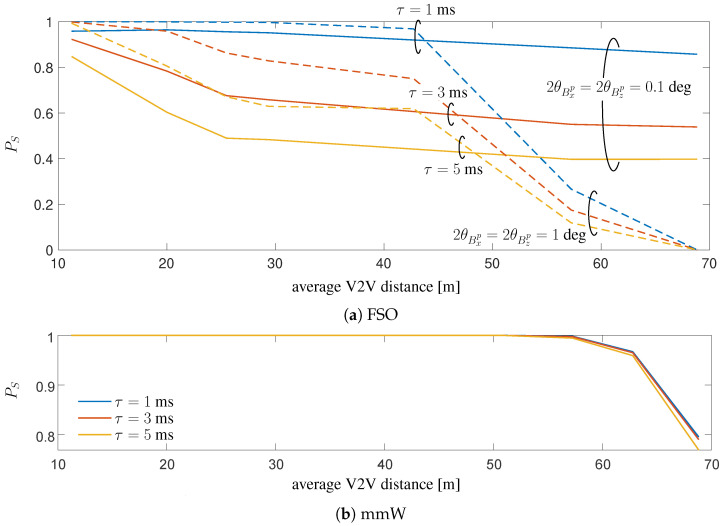
Service probability of the proposed sensor-aided method versus distance (obtained from the time gap Δt) on trajectory S2 for different values of delay τ. (**a**) FSO V2V for both HD and LD configurations, (**b**) mmW V2V for mmWLD ($N_{a}=32\;,N_{r}=8$).

Referring to the results in Figure 8 and Figure 9, the latency is a significant V2X degradation impairment for the proposed sensor-aided technique, with a meaningful detrimental impact for FSO, as the transmitted power is confined in a very restricted area. This impact is deeply analyzed in Figure 10, where the service probability $P_{S}$ for FSO is evaluated versus the average V2V distance for S2. This probability is computed as $P_{S}=\mathrm{Prob}\left(\mathrm{SNR}>\Gamma \right),$ with Γ=10 dB for both technologies in order to guarantee a fair comparison (This value for FSO corresponds to the threshold value that guarantees a Bit Error Rate (BER) ≤ 1.3×10${}^{-2}$ for a Return to Zero On-Off Keying (RZ-OOK) modulation, which is the standard value for employing a 20%-overhead Hard Detection-Forward Error Correction Code (HD-FEC) for optical communications [68,69] ). According to Figure 10a, we can conclude that in a complex scenario as S2, where the vehicle dynamic is over-complicated by the presence of multiple road turnings across a contour, a relatively reliable FSO V2V communication is enabled only by a very low-latency control signaling (1 ms, as foreseen by 5G system) and for distances up to 30 m (for which $1-P_{S}\leq 10^{-2}$). By contrast, mmW appears to be much more robust to delay, as can be expected from the use of less directive beams and observed from Figure 10b. It is to be noticed that for distances greater than 55 m, the reduction of $P_{S}$ for *mmW LD* is to be attributed to the high path loss.

**Figure 11:**
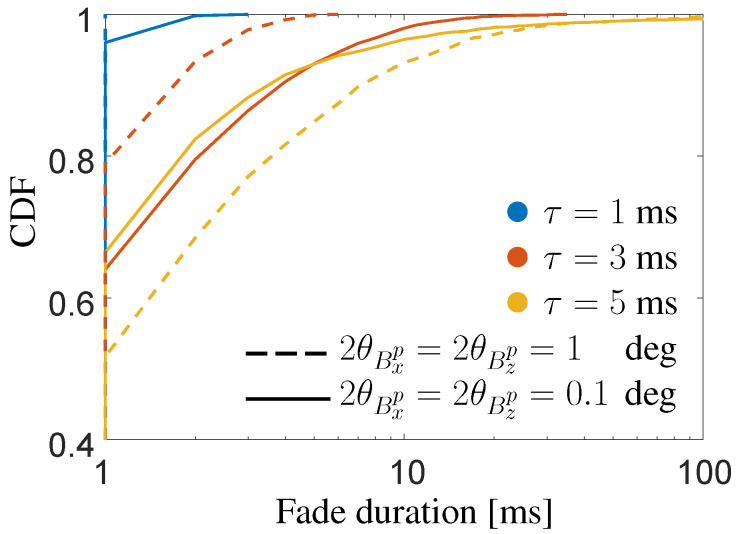
CDF of the FDD in the proposed sensor-aided method in scenario S2 for both FSO configurations and three different values of update delay τ.

Besides the analysis of the service probability, in eV2X applications it is important to characterize the outage events. In this regard, we report the distribution of the outage event, evaluating not only its occurrence but also its duration in terms of FDD. The fade duration is defined the time interval by which the SNR persists to be below 10 dB (SNR<Γ). It can be proved that the fade duration is negative exponentially distributed, with the CDF shown in Figure 11 for both FSO configurations in S2. Once again, we further highlight the importance of sharing timely information to achieve a virtually continuous beam-tracking. It is to be noticed that the standalone analysis of FDD might lead to misleading conclusions on the use of wide/narrow beams. Considering the curves for a delay of 5 ms, one may conclude that a HD system is more performing than a LD one, as it has shorter fade events. Clearly, this is not valid as it is extremely more challenging to use narrow beams rather than large ones for the considered applications, as largely motivated and proved in this article. However, this unexpected behavior is to be attributed to the susceptibility of narrow beams, which produces a jumping-like radiating spot at the receiver side. We found that in this specific case the number of fade events for the *FSO HD* system (yellow solid line) is 54% higher than for *FSO LD* (yellow dashed line), leading us to conclude that a narrow beam system has a higher number of fade events of shorter duration each.

**Figure 12:**
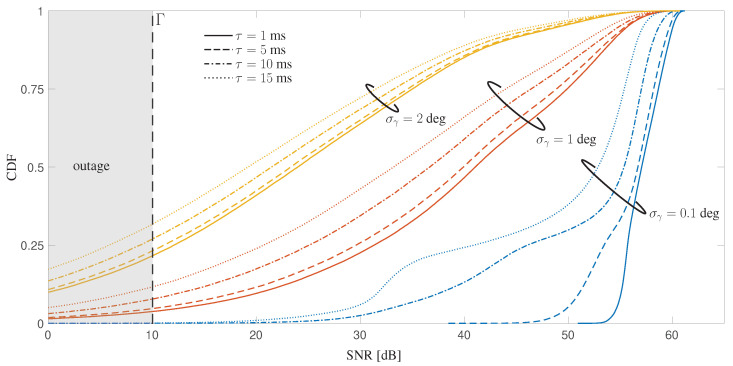
CDF of the SNR for different angular accuracy $\sigma_{\gamma }$ on scenario S2 of the proposed sensor-aided method. The results are plotted for mmWHD for different values of delay τ (vehicle time gap Δt=1 s).

To summarize, V2X over mmW, and especially *mmW HD* (180 × 90 antenna elements), guarantees a seamless service (no outage was observed for the considered scenario), and it is a valid candidate for high-speed V2X. However, it is to be noticed that the angular accuracy strongly impacts on the overall performance. In case of inaccurate sensors or orientation estimates, such that $\sigma_{\gamma }$ is in the order of 1–2 deg, the best performing *mmW HD* V2V system experiences a meaningful performance degradation, as shown in Figure 12 for a reference vehicle gap Δt=1 s and different delay values. This result highlights the need for a very precise pose estimation that can be achieved either by single precise sensor or by proper data fusion algorithms of on-board IMU and external sensors.

## 6. Concluding Remarks and Future Directions

This paper introduces a Cooperative Intelligent Transportation System (C-ITS) architecture for Vehicle-to-Anything (V2X) communications based on either millimeter-Wave (mmW) or Free-Space Optics (FSO) technologies. In order to keep the extremely narrow beams of both FSO and mmW aligned, and thus to guarantee a seamless connectivity, we propose to exploit the data gathered from the numerous on-board sensors, presently largely installed on vehicles, integrating typical mechanical information with telecommunication apparatus. Each vehicle first estimates its own pose from these sensor data, and then exchanges this information with all the others so that all vehicles in the network acquire a full knowledge of the system geometry. In the proposed architecture, this sensor data exchange among vehicles is over a parallel low-rate control channel, so that vehicles can take full advantage of the multi-gigabit V2X link, i.e., either FSO- or mmW-based, to meet the stringent low-latency and high data-rate requirements of advanced C-ITS services.

Numerical simulations based on a general 3D geometrical model and on realistic vehicles motions over a winding road confirm the feasibility of the proposed sensor-aided mmW- or FSO-based C-ITS architecture, demonstrating remarkable improvements with respect to conventional beam sweeping scheme and providing an alternative to the onerous exhaustive beam search. The mmW and FSO solutions have been compared in high and low directivity configurations to assess the robustness of the proposed technique with respect to the beam dimension and pose estimation errors. We verified the intuition that misalignment errors have a more detrimental impact on narrow beams by assessing the performance degradation in terms of Cumulative Distribution Function (CDF) of the Signal-to-Noise Ratio (SNR). This analysis has been used to statistically characterize the outage events in terms of occurrence probability and distribution of fade events. In particular, the main takeaways are: (*i*) mmW and FSO technologies are attractive candidates for V2X communications, but the latter solution is viable only if precise pose information is available; (*ii*) under the same power and bandwidth settings, FSO is potentially able to provide superior performance due to its highly directive laser beams, but it is extremely sensitive to pointing errors; (*iii*) in the presence of complex motion scenarios, it is convenient to employ technologies that rely on wider beams, such as those achieved by mmW technology; (*iv*) mmW is in principle capable of attaining the capacity of FSO, but only by deploying thousands of antenna elements, e.g., 180×90, which is not feasible considering today’s commercial hardware.

As a final consideration, in this article we proposed the integration of two engineering domain that might have been considered ad standalone up to now. In our view, the evolution of connected mobility and related service requirements calls for a new paradigm of V2X communications. We deem it is highly recommended to support the telecommunication apparatus with vehicle kinematics data, using sensors’ information for both advanced driving functionalities and telecommunication purposes. This is the leading idea behind the proposed approach, whose feasibility has been studied herein.

Future works could include the evaluation of the impact of adverse atmospheric conditions in the performance of the FSO-based link. It is also crucial to integrate into the proposed mmW-based solution the design of hybrid beamforming techniques, which are mandatory in practice to handle a massive number of antenna elements at each vehicle. Furthermore, beside the conventional beamforming considered in this paper, more advanced beamforming techniques needs to be considered, including more refined interference rejection for simultaneous communication between different V2X agents. Finally, the overall theoretical discussion mandatory needs to be supported by a hardware prototype in order to demonstrate that the proposed C-ITS architecture is not only an attractive research topic offering numerous theoretical insights, but mainly a practical solution for V2X systems. This last point is already object of ongoing investigations. 

## Figures and Tables

**Table 1:** Simulation parameters.

Parameter	Symbol	Value	Parameter	Symbol	Value
Tx power	$P_{\mathrm{tx}}$	1 mW	Signal bandwidth	*B*	2.16 GHz
Update delay	τ	1–15 ms	Sensor sampling freq.	$f_{\mathrm{data}}$	1 kHz
Vehicle height	$h_{1},h_{2}$	1.5 , 1.7 m	Vehicle length	$\ell_{1},\ell_{2}$	4.7 m
Position error	$\sigma_{p}$	10 cm	Angular error	$\sigma_{\gamma }$	0.1, 1, 2 deg
**mmW-specific**					
Frequency	*f*	60 GHz	Path Loss exponent	κ	2
Shadowing std. dev.	$\sigma_{\mathrm{dB}}$	0 dB	Noise floor	$N_{fl}$	−174 dBm/Hz
Rx Noise Figure	NF	6 dB	Pattern parameters	b, c	1.8 , 1.6
**FSO-specific**					
Tx Laser wavelength	λ	1550 nm	Rx aperture (single PD)	$A_{\mathrm{rx}}$	1 cm${}^{2}$
Rx array diameter	*D*	10 cm	Rx array height	*H*	5 cm
PD responsivity	ρ	0.9 A/W	Optical filter bandwidth	Δλ	50 nm
Solar irradiance	$I_{b}$	5.58 $W/m^{2}$	Noise Equivalent Power	NEP	20 $\mathrm{pW}/\sqrt{\mathrm{Hz}}$ [65]
**Trajectories**—according to Equation (29)					
Vehicle speed	*v*	50 km/h	Angular vel.	$\omega_{0}$	0.04π rad/s,
Horizontal angular vel.	$\omega_{h}$	0.8π rad/s	Vertical angular vel.	$\omega_{v}$	0.08π rad/s,
Horizontal amplitude	$A_{h}$	0.1 m	Vertical amplitude	$A_{v}$	10 m
Radius	$r_{0}$	$v/\omega_{0}$ m	Vehicle time gap	Δt	0.5–5 s

**Table 2:** Parameters of mmW and FSO beams for *LD* and *HD* configurations.

mmW					FSO	
	$N_{a}$	$N_{r}$	$\Delta \alpha_{-3\mathrm{dB}}$	$\Delta \beta_{-3\mathrm{dB}}$	$2\theta_{B_{x}^{p}}$	$2\theta_{B_{z}^{p}}$
*LD*	32	8	∼34.5deg	∼12.5deg	1deg	1deg
*HD*	180	90	∼9deg	∼1.12deg	0.1deg	0.1deg
